# Chromatin-mediated regulators of meiotic recombination revealed by proteomics of a recombination hotspot

**DOI:** 10.1186/s13072-018-0233-x

**Published:** 2018-10-29

**Authors:** Aaron J. Storey, Hsin-Ping Wang, Reine U. Protacio, Mari K. Davidson, Alan J. Tackett, Wayne P. Wahls

**Affiliations:** Department of Biochemistry and Molecular Biology, University of Arkansas for Medical Sciencs, 4301 West Markham Street (Slot 516), Little Rock, AR 72205-7199 USA

**Keywords:** Homologous recombination, Meiosis, Histones, Chromatin remodeling, Mass spectrometry, Proteomics, *Schizosaccharomyces pombe*

## Abstract

**Background:**

Meiotic recombination hotspots control the frequency and distribution of Spo11 (Rec12)-initiated recombination in the genome. Recombination occurs within and is regulated in part by chromatin structure, but relatively few of the many chromatin remodeling factors and histone posttranslational modifications (PTMs) have been interrogated for a role in the process.

**Results:**

We developed a chromatin affinity purification and mass spectrometry-based approach to identify proteins and histone PTMs that regulate recombination hotspots. Small (4.2 kbp) minichromosomes (MiniCs) bearing the fission yeast *ade6*-*M26* hotspot or a basal recombination control were purified approximately 100,000-fold under native conditions from meiosis; then, associated proteins and histone PTMs were identified by mass spectrometry. Proteins and PTMs enriched at the hotspot included known regulators (Atf1, Pcr1, Mst2, Snf22, H3K14ac), validating the approach. The abundance of individual histones varied dynamically during meiotic progression in hotspot versus basal control MiniCs, as did a subset of 34 different histone PTMs, implicating these as potential regulators. Measurements of basal and hotspot recombination in null mutants confirmed that additional, hotspot-enriched proteins are *bona fide* regulators of hotspot activation within the genome. These chromatin-mediated regulators include histone H2A-H2B and H3-H4 chaperones (Nap1, Hip1/Hir1), subunits of the Ino80 complex (Arp5, Arp8), a DNA helicase/E3 ubiquitin ligase (Rrp2), components of a Swi2/Snf2 family remodeling complex (Swr1, Swc2), and a nucleosome evictor (Fft3/Fun30).

**Conclusions:**

Overall, our findings indicate that a remarkably diverse collection of chromatin remodeling factors and histone PTMs participate in designating where meiotic recombination occurs in the genome, and they provide new insight into molecular mechanisms of the process.

**Electronic supplementary material:**

The online version of this article (10.1186/s13072-018-0233-x) contains supplementary material, which is available to authorized users.

## Background

In meiosis, cells express the broadly conserved Rec12/Spo11 (topoisomerase II-like) protein which, along with other components of the basal meiotic recombination machinery, catalyzes the formation of dsDNA breaks (DSBs) that initiate meiotic recombination [1]. While meiotic recombination can occur anywhere along chromosomes, it is clustered at hotspots that regulate its frequency and distribution in the genome [2–4]. As with all DNA-dependent processes (e.g., transcription), the basal meiotic recombination machinery must gain access to its substrates within, and is therefore regulated in part by, chromatin structure.

Sequence-specific DNA binding proteins, such as the Atf-Pcr1 heterodimer, Bas1, and Prdm9, regulate hotspots [3, 5–7]. They trigger directly or indirectly posttranslational modifications (PTMs) of histones that help position recombination through the modulation of chromatin structure [8–11]. Individual species can have many hotspot-activating protein–DNA complexes [12–15], and to the extent tested, their regulatory functions are conserved in other species [16]. Similarly, histone PTMs, such as acetylation (e.g., H3K9ac and H3K14ac), ubiquitination (e.g., H2BK123ub), and methylation (e.g., H3K4me3 and H3K36me3), contribute to hotspot activation. The removal of PTM acceptor residues or the enzymes that place these marks, such as the histone acetyltransferase Gcn5, the E3 ubiquitin ligase Bre1/Brl1, and Set1 methyltransferases (of which Prdm9 is a member), and the removal of ATP-dependent chromatin remodeling enzymes such as Snf22, affect the distribution of recombination at hotspots (e.g., [17–21]). Differences between species reflect variations on the theme. For example, there are differences in dependence on Set1 methyltransferase activity (e.g., [19–21]), and in some species, a DNA binding domain targets the enzyme to the chromosome, whereas in other species there is no DNA binding domain, so the enzymatic activity must be recruited indirectly by other factors (e.g., [19, 22]).

Interestingly, all factors known to help position meiotic recombination at hotspots display context variable penetrance, indicating that they must function in concert (together or sequentially) with other, yet unidentified factors to promote recombination locally [3, 23, 24]. For example, while the hotspot-activating Atf1-Pcr1 heterodimer [12, 25] binds to most of its *M26* DNA sites in the genome [26, 27], only about one quarter of those protein–DNA complexes activate hotspots [5]. This property also applies for other sequence-dependent hotspots [14, 28, 29], for regulatory histone PTMs [20, 30], and for “open” chromatin (as judged by sensitivity of DNA within chromatin to nucleases) [31, 32]. Additional regulatory complexity comes from the fact that chromatin morphogenesis involves an ordered sequence of reactions whose detection requires the ability to analyze discrete time points within highly synchronous populations of meiotic cells. For example, the hotspot-dependent acetylation of histone H3 residues that help to position recombination is induced transiently in meiosis—and falls substantially before the time when Rec12/Spo11 catalyzes the formation of DSBs [17, 20]. To further complicate matters, eukaryotes contain vast numbers of chromatin remodeling factors and histone PTMs, relatively few of which have been interrogated for a role in regulating meiotic recombination.

**Fig. 1:**
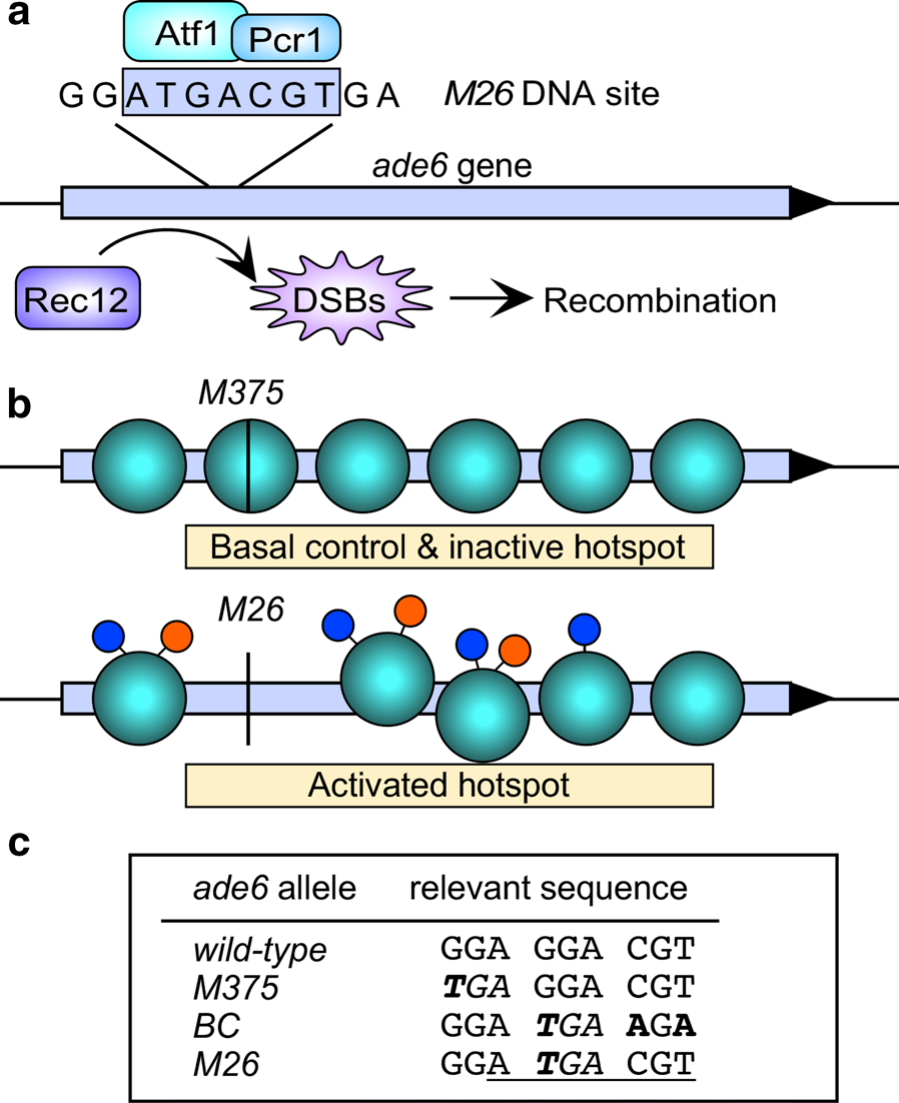
Features of the *ade6*-*M26* meiotic recombination hotspot. **a** Binding of Atf1-Pcr1 heterodimer to an *M26* DNA sequence motif promotes the catalysis of recombination-initiating DSBs by Rec12 (Spo11). **b** Hotspot-specific, meiotically induced chromatin remodeling, involving histone PTMs (lollipops) and the displacement of nucleosomes (ovals), generates access to DNA and potential docking moieties for the basal recombination machinery and its catalytic subunit, Rec12 (Spo11). **c** Sequences of alleles used in this study. Each allele contains bp substitutions (bold) that create a stop codon (italics) in the *ade6* ORF. Hotspot alleles contain an *M26* DNA site (underlined) to which the Atf1-Pcr1 heterodimer binds

In this study, we sought to define as comprehensively as possible the local epiproteome (proteins and histone PTMs) of a well-defined, DNA sequence-dependent meiotic recombination hotspot, *ade6*-*M26* of fission yeast (Fig. 1). Binding of the Atf1-Pcr1 (Mts1-Mts2) heterodimer [25] to an *M26* DNA sequence motif (5′-ATGACGT-3′) [33] activates the hotspot [12, 25, 26, 34]. This protein–DNA complex triggers hotspot-specific chromatin remodeling that promotes the local catalytic activity of the basal recombination machinery [17, 20, 35]. A control allele that lacks the *M26* DNA site (*M375* or *BC*) lacks hotspot activity but supports basal levels of recombination, permitting one to determine whether a given factor (e.g., protein or histone PTM) is specific to hotspot activation or affects more generally the basal recombination machinery [12, 17, 20, 25, 34, 36–38]. Since one can induce highly synchronous meiosis in large cultures of fission yeast [39], we reasoned that we could use high-resolution, high-sensitivity mass spectrometry (MS) to discover dynamic changes in protein occupancy and histone PTMs that occur at sequential time points of meiosis.

We report the development of a way to purify discrete, unit-length segments of chromatin to near homogeneity, the discovery of numerous, dynamic changes in protein occupancy and histone modifications at the *M26* hotspot, and confirmation that newly identified, broadly conserved, hotspot-enriched factors are *bona fide* regulators of hotspots.

## Results

### A minichromosome (MiniC) approach to define the local epiproteome

We first applied published methods for chromatin affinity purification with mass spectrometry (ChAP-MS) [40] and CRISPR-ChAP-MS [41] to enrich for chromatin fragments from the *ade6* locus in the genome. Following optimization, we were able to enrich *ade6* chromatin fragments up to 100-fold, relative to those from loci elsewhere in the genome, but we deemed this level of purification inadequate to meet our goals (even a 1000-fold enrichment would be inadequate). The reason is straightforward and is germane to all such studies. If a chemically cross-linked, 12,500-kbp genome is sheared into chromatin fragments approximately 1 kbp in length and if the target fragment is enriched 1000-fold, then greater than 90% of proteins in the purifications would come from genomic regions other than the target locus of interest. To increase the likelihood of discovering factors associated specifically with hotspot chromatin, relative to basal control chromatin, we needed to increase substantially the degree of purification.

The chromatin structure of *ade6* in a plasmid is like that in the chromosome [42], and episomes have been used successfully to enrich chromatin for analyses of its components [43]. We reasoned that small, circular minichromosomes (MiniCs), without any *E. coli* plasmid backbone, would provide several advantages. First, since they are extrachromosomal elements, there would be no need to shear chromosomes to liberate the target locus. Second, the omission of shearing would obviate the need for prior chemical cross-linking of proteins to DNA and would streamline the process, allowing us to purify chromatin rapidly under native conditions. Third, the large differences in size (and other biophysical characteristics) between MiniCs and chromosomes might improve partitioning, and hence degree of enrichment, during purifications. Fourth, since the MiniCs are of unit length, they would lack the heterogeneity intrinsic to sheared chromatin and would, correspondingly, provide a more discrete readout of associated factors.

**Fig. 2:**
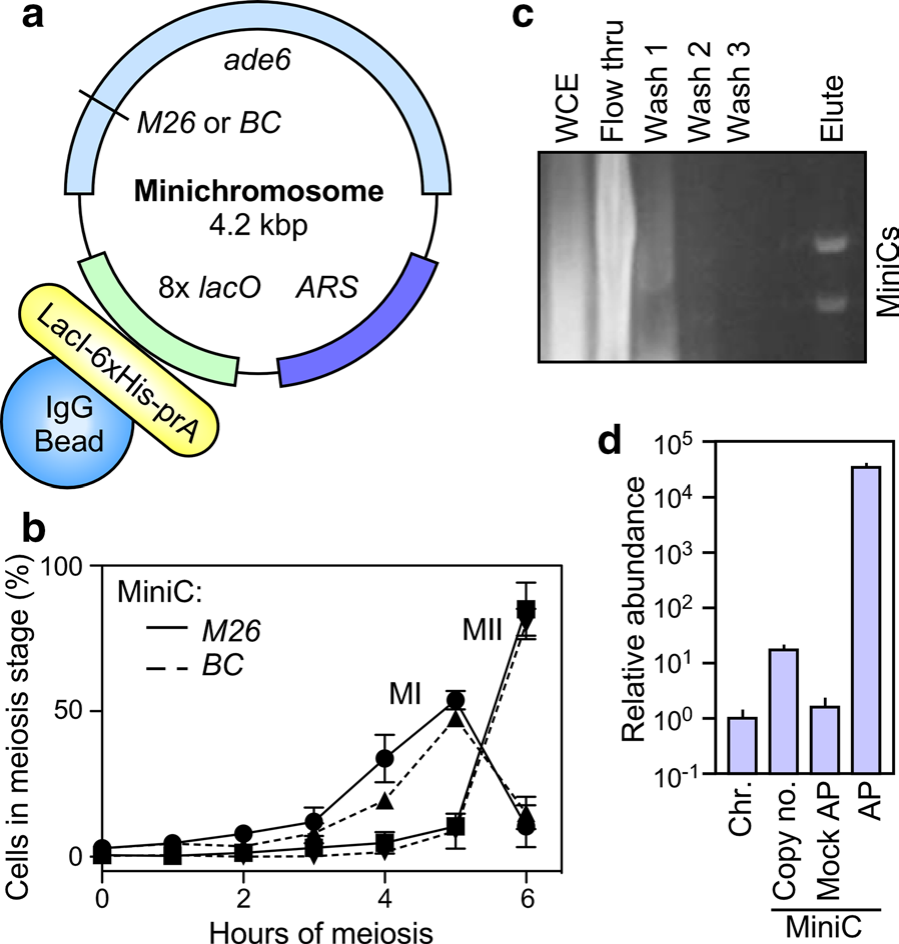
Purification of minichromosomes (MiniCs) from highly synchronous meiosis. **a** Structure of MiniCs. Recombination hotspot (*M26*) and basal control (*BC*) MiniCs contain different alleles of the *ade6* gene, a fission yeast origin of replication (*ARS*) and copies of the *LacO* DNA site for affinity purification. **b** Efficiency and synchrony of induced meiosis. Plots show the frequencies of cells undergoing the first meiotic division (*MI*, 2 nuclei) and having completed the second meiotic division (*MII*, 3–4 nuclei) in strains harboring the indicated MiniCs. **c** The indicated samples of chromatin from steps of purification were deproteinized, and their DNAs were analyzed by agarose gel electrophoresis (*WCE*, whole-cell extract). **d** MiniC copy number and degree of enrichment; note log scale. The abundance of *ade6* DNA in the chromosome (*Chr*) or in the MiniC (with chromosomal *ade6* deleted) was determined by qPCR, relative to the *act1* locus, and those values were normalized relative to single-copy *ade6* in the chromosome. Affinity purifications (*AP*) employed LacI-6xHis-prA; mock AP samples were processed identically, but lacked LacI-6xHis-prA. In this figure and others, plots with error bars are mean ± SD from three or more biological replicates

We therefore constructed MiniCs that harbor only three elements: a fission yeast origin of replication (autonomously replicating sequence, *ARS*), the *ade6* gene, and eight copies of the *LacO* DNA site (Fig. 2a). Versions of the MiniC bearing the hotspot (*M26*) and basal control (*M375* or *BC*) alleles of *ade6* differ at only two base pairs and by the presence or absence of the *M26* DNA site to which the hotspot-activating Atf1-Pcr1 heterodimer binds (Fig. 1c). At only 4.2 kbp in size, these MiniCs can harbor a maximum of about 25 nucleosomes, although the actual number is likely lower due to nucleosome-depleted regions (NDRs) in the *ade6* promoter and the *ARS* [17, 32]. Using the minimum possible size for MiniCs maximized the likelihood that we could detect highly localized changes in chromatin, such as histone PTMs that occur on only one or a few nucleosomes at or close to the hotspot DNA sequence motif.

The hotspot (*M26*) and basal control (*M375*) alleles of *ade6* each contain a stop codon near the 5′ end of the *ade6* ORF (Fig. 1c) [44]. Since these stop codons are reportedly suppressible by the nonsense codon suppressor *sup9e* [45, 46], we reasoned that we could use selection for adenine prototrophy in cells expressing *sup9e* to maintain selection for the MiniCs within a host strain deleted for the chromosomal *ade6* gene. This would be advantageous because it would obviate the need for an additional selectable marker, keeping the MiniCs as small as possible. Unfortunately, we encountered two unexpected problems.

First, the *sup9e* strain [46] did not contain *sup9e*, as initially defined [47]. We discovered subsequently that this strain harbors a previously uncharacterized suppressor, *sup35*-*F592S* [48]. Second, while both the real *sup9e* and *sup35*-*F592S* were effective suppressors of adenine auxotrophy caused by the *M26* allele, neither one effectively suppressed *M375* [48]. In short, we lacked a means to select for maintenance of MiniC-*M375*. We therefore created a new basal control allele, *ade6*-*BC*, that harbors the same (suppressible) stop codon as *ade6*-*M26*, but that lacks the DNA binding site for the hotspot-activating Atf1-Pcr1 heterodimer (Fig. 1c). The new *BC* control allele behaved like the *M375* control when tested for recombination in the genome, and it supported selection for the MiniC as effectively as did *M26*. Thus, we established a way to compare directly recombination hotspot and basal recombination control alleles within MiniCs.

### Efficient, highly synchronous meiosis in strains harboring MiniCs

Thermal inactivation of the Pat1-114^ts^ protein (a key repressor of meiosis) supports the induction of synchronous meiosis in *S. pombe* (e.g., [39, 49–52]). In such meioses, DSBs appear between about 3 and 4 h (after which they are repaired) and the subsequent two meiotic divisions are completed by about 6 h. Our strains harbored MiniCs and the *sup35*-*F592S* mutation required for their maintenance, which might affect the efficiency of meiotic induction and progression. To test this, we used our version (see Methods) of standard induction protocols and monitored the timing of the two meiotic divisions. Strains harboring the hotspot and basal control MiniCs were highly proficient for meiosis, and importantly, they each displayed equivalent timing (Fig. 2b). This established that the biological samples are well matched, temporally and developmentally, supporting direct, reciprocally controlled comparisons of samples at discrete time points of meiosis. The timing of events, which was like that reported in other studies, also established the time window for subsequent analyses of chromatin components.

### Purification of MiniCs to near homogeneity

We purified MiniCs from whole-cell extracts under native, stringent (300 mM KCl) conditions by affinity capture, taking advantage of the high-affinity, multivalent interactions between Lac repressor (LacI) and *LacO* DNA sites. To generate the affinity capture reagent, we expressed and purified a fusion protein that contains LacI, a hexahistidine (6xHis) tag, and protein A (prA) (Additional file 1: Fig. S1). The prA moiety of LacI-6xHis-prA binds with high affinity to IgG, permitting us to capture the fusion protein (and its MiniC cargo) using IgG that is bound covalently to magnetic beads. Cells were lysed under cryogenic conditions, LacI-6xHis-prA was added to the thawed extract, magnetic IgG-Dynabeads were added, and sequential iterations of magnetic capture and washing with native buffer were carried out; then, the proteins were eluted from the chromatin. In each experiment, the degree of enrichment was monitored by extracting DNA from an aliquot of the chromatin, followed by qPCR to measure the abundance MiniC DNA (*ade6*) relative to DNA from the genome (*act1*).

The qPCR analyses of unfractionated material revealed that MiniCs are maintained at a copy number of about 20 per cell (Fig. 2d). Following optimization of conditions for affinity capture (e.g., Additional file 1: Fig. S2), samples of DNA from within chromatin at sequential steps of purification were analyzed by agarose gel electrophoresis (Fig. 2c). Unfractionated cell extract yielded a broad smear of signal derived from chromosomal DNA that was sheared by the forces used to disrupt the cells. Those chromosomal DNA fragments were undetectable in the purified sample, which contained discrete bands corresponding to MiniCs. Analyses of the abundance of MiniC DNA by qPCR revealed that we had achieved nearly a 100,000-fold enrichment of MiniC chromatin, relative to fragments of chromatin from the genome (Fig. 2d). That enrichment strictly required the presence of the LacI-6xHis-prA moiety used to capture the MiniCs. We conclude that we can purify MiniCs to near homogeneity and that greater than 95% of the proteins recovered by these purifications come from their association with the MiniCs.

This does not mean that all of the recovered proteins were components of, or were interacting with, MiniCs in vivo. As is the case for every affinity purification and immuno-purification experiment that is conducted, many proteins associate artifactually following homogenization of cell contents. The magnetic bead-adsorbed MiniCs are, for example, essentially mixed bed ion exchange matrices to which other positively and negatively charged proteins can bind. However, our well-matched hotspot and basal control alleles (Fig. 1c) provided a way to focus on factors that are enriched preferentially at the hotspot (candidate regulators) and to discount many of the proteins that interact artifactually with both hotspot and basal control MiniCs ex vivo.

### Mass spectrometry and normalization of datasets

To discover factors enriched at the hotspot, we conducted affinity purifications of MiniC chromatin from four time points of synchronous meiosis spanning from induction (0 h) to the time (3 h) just before the first meiotic division (4 h, Fig. 2b). In each experiment, we compared hotspot (*M26*) to basal control (*BC*). We used three independent biological replicates except for the 1-h time point, for which there were duplicates. Proteins in each sample were identified and quantified by liquid chromatography–tandem mass spectrometry (LC–MS/MS) analyses of tryptic peptides using a Thermo Orbitrap Fusion Tribrid mass spectrometer. For broader depth of coverage, the peptides were first fractionated by LC under basic conditions (bLC), and then, those fractions were concatenated (*e.g*., by pooling fractions 1, 16, and 31; 2, 17, and 32). In total, 437 concatenated fraction pools were each analyzed by LC–MS/MS, which identified 21,362 unique peptides corresponding to 2721 proteins. The abundance of each protein was determined using intensity-based absolute quantification (iBAQ), and the abundance of a given peptide harboring one or more histone PTMs was determined relative to all occurrences of that peptide [53–55]. Primary data files from which the iBAQ values were obtained are available in the posted dataset, which has been deposited in the ProteomeXchange database (see Availability of data and materials).

**Fig. 3:**
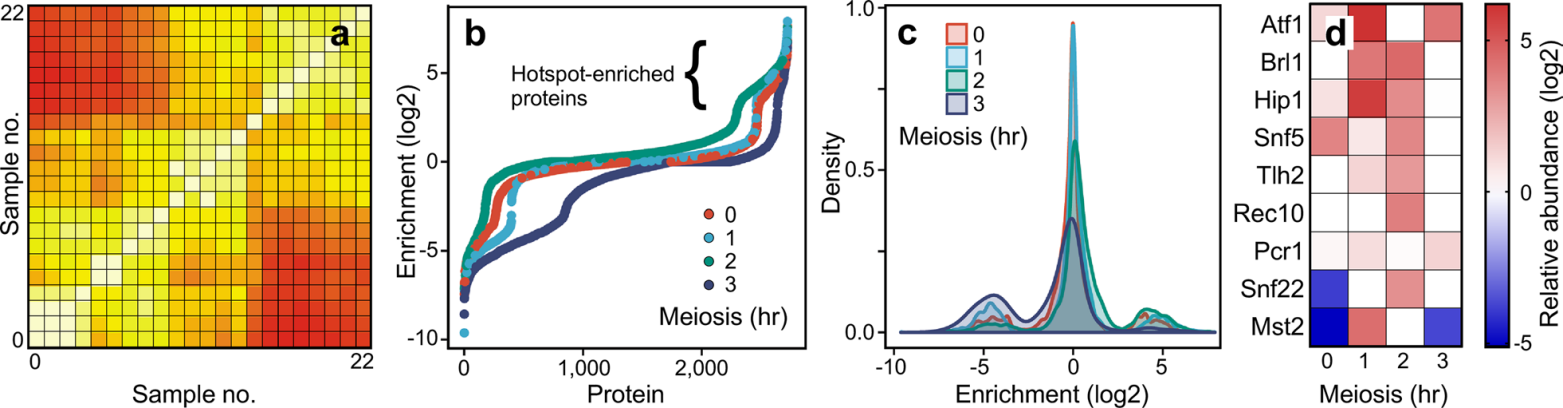
Identification of hotspot-enriched proteins during meiosis. Mass spectrometry was used to determine the abundance of proteins from affinity purifications of hotspot and basal control MiniCs at four time points of meiosis. **a** Heat map shows Pearson correlation coefficient (*r*) for each pairwise comparison of all experimental conditions and all biological replicates; *r* values range from 0.6 (darkest red) to 1.0 (white) (see Additional file 1: Fig. S4 for additional details). **b** Relative abundance (hotspot vs basal control) of every detected protein at each time point of meiosis; note log2 scale. **c** Density plots show changes in relative protein abundance (*X*-axis) versus sum of total protein abundance (*Y*-axis) over time. **d** Heat map shows log2 relative enrichment of proteins in hotspot versus basal control for a subset of the chromatin-associated/nuclear proteins that were enriched at one or more time points in the hotspot sample (for a more extensive list, see Additional file 1: Table S2)

The large number of biological samples required the processing of affinity purifications and MS analyses in batches, which affected the absolute yield of proteins as measured precisely by MS (Additional file 1: Fig. S3a). To account for such effects, we normalized the protein abundance values within each biological sample relative to the median value of all experiments (i.e., we controlled for total protein abundance in each sample) (Additional file 1: Fig. S3b). We determined the Pearson correlation coefficient (*r*) between all pairwise comparisons of samples, taking into account the abundance of each protein in each sample (Fig. 3a; Additional file 1: Fig. S4). Interestingly, hierarchical clustering revealed that the greatest differences tended to group according to experimental batch (compare Additional file 1: Figs. S4 to S3), indicating that batch effects persist to some extent even after normalization, which frequently occurs in high-throughput data [56]. Thus, the binary nature of MS-based discovery (protein detected or not), nonzero detection limits for continuous variables (iBAQ values), and inter-batch variability affected the precision of measurements. For these reasons, we used the sum of normalized iBAQ values to determine relative abundance in hotspot versus basal control. To eliminate division errors and infinity ratios, if one sample in a matched pair (hotspot or control) had a zero iBAQ value for a given protein, then that protein was assigned a nonzero iBAQ value (5000) corresponding to the bottom of the detection range.

### MiniC-AP-MS reveals known and candidate regulatory proteins

To identify candidate regulatory proteins, we compared the relative abundance (hotspot versus basal control) for each protein detected at each time point of meiosis. While majority of the proteins were of similar abundance in hotspot and basal control, subsets of proteins were enriched in either the hotspot or the basal control (Fig. 3b). This is consistent with our expectation that while there would be many proteins associated with MiniCs, only a subset would be specific to the hotspot. The differences in relative protein abundance increased with progression through meiosis (revealed by the decrease in central peak height and broadening of the overall distribution shown in Fig. 3c), as one might expect from a cascade of downstream events triggered by the presence (or absence) of the hotspot-activating Atf1-Pcr1-*M26* protein–DNA complex.

Given that hotspot activation likely involves an ordered sequence of potentially transient events within chromatin that culminates in the formation of Rec12 (Spo11)-catalyzed DSBs, we constructed a list of proteins that were enriched at least twofold at the hotspot, for one or more of the meiotic time points, and that were annotated as being either nuclear or associated with chromatin (see Fig. 3d for examples and Additional file 1: Table S2 for a more extensive list). Among these proteins were those already known to regulate the hotspot—including both subunits of the primary activating module (Atf1-Pcr1 heterodimer) [12, 25], a histone-modifying enzyme (acetyltransferase Mst2) [57], and an ATP-dependent chromatin remodeler (Snf22) [17] (Fig. 3d). The identification of these proteins previously implicated in hotspot regulation strongly supports the idea that the *M26*-bearing MiniC serves as a good surrogate for its genomic counterpart and that the *M26* allele remains an active hotspot in this context. We conclude that our MiniC-AP-MS approach can identify *bona fide* regulators of recombination hotspots.

Our screen for hotspot-enriched factors also uncovered a large collection of additional, candidate regulatory proteins (Fig. 3d, Additional file 1: Table S2 and publically available datasets), including many that are known to affect chromatin structure. These included histone code writers, readers and erasers, histone chaperones, and ATP-dependent chromatin remodelers. We describe several of these candidates in greater detail below—and we show that they are *bona fide* regulators of meiotic recombination hotspots within chromosomes.

### Changes in histone occupancy

**Fig. 4:**
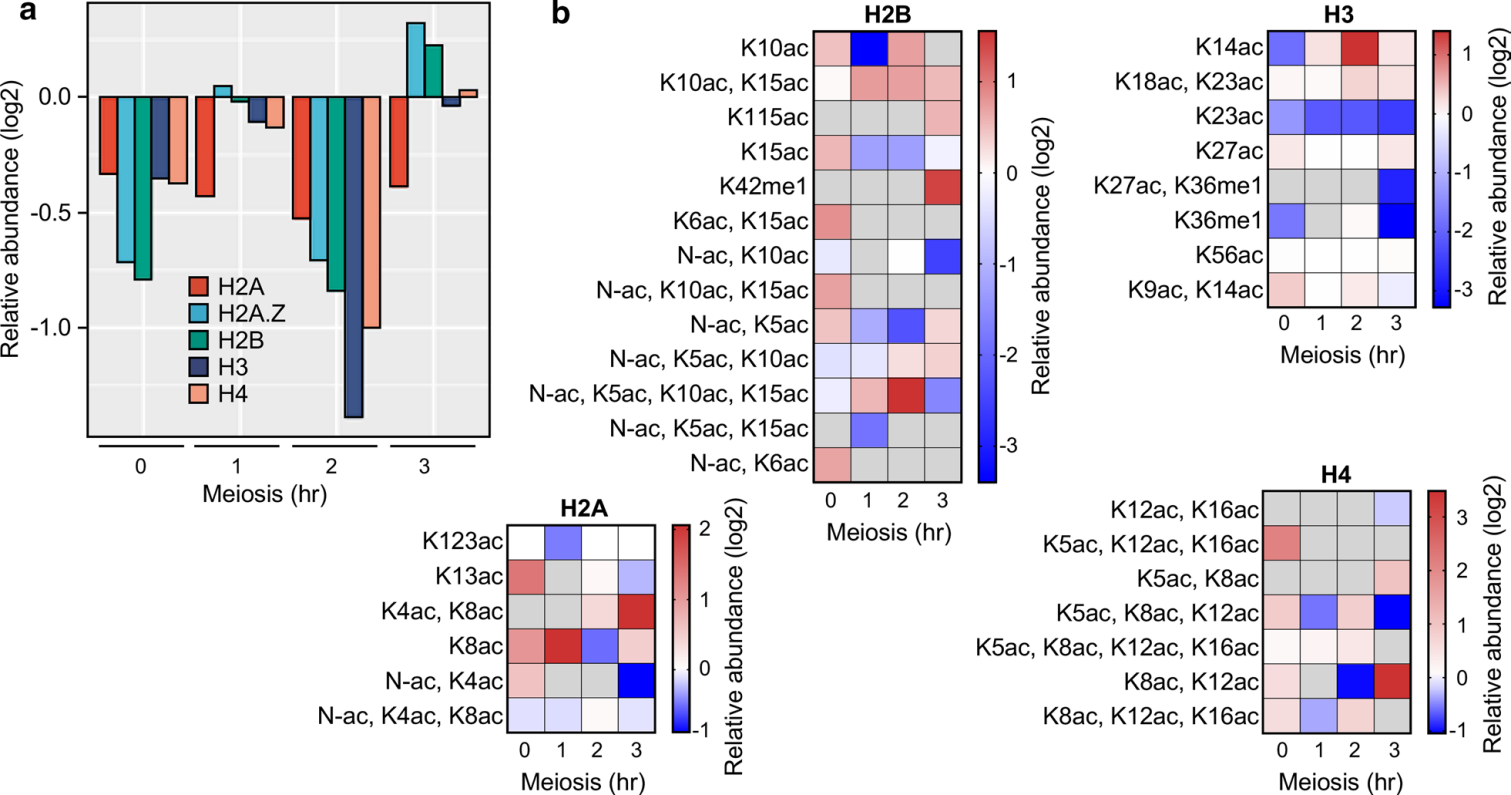
Differences in abundance of histone subunits and histone PTMs at hotspot and basal control in meiosis. Protein abundance was from iBAQ values; precursor intensity values for histone peptides with and without modifications were used to quantify the abundance of histone PTMs. **a** Relative abundance of histone subunits; negative (log2) values indicate reduction in histone occupancy within hotspot MiniCs relative to basal control MiniCs. **b** Relative abundance of all histone PTMs detected; *gray* cells indicate that the modified peptide was not detected in one or both samples (hotspot or control). Note that the majority of peptides with a PTM harbor multiple PTMs, revealing combinatorial complexity not detectable by ChIP

As expected for purified chromatin, histones were among the most abundant proteins detected. There were dynamic changes in the relative occupancy of histones H2A, H2A.Z, H2B, H3, and H4 between hotspot and basal control MiniCs (Fig. 4a). The abundance of the individual histones in hotspot MiniCs was lower (57–81% of control) at the 0-h time point, became more equal at 1 h, fell again at 2 h (39–69% vs control), and became more equal at 3 h. It should be emphasized that these data and those for histone PTMs (next section) reflect the population-average occupancy of histones and PTMs throughout the 4.2-kbp minichromosome and hence cannot tell us about occupancy precisely at the hotspot. Nevertheless, the observed changes are consistent with the previous reports of *M26*-dependent chromatin remodeling at the *ade6*-*M26* hotspot (as judged by sensitivity of DNA in chromatin to MNase) [17, 57] and changes in chromatin structure at *M26* DNA sites elsewhere [58]. The newly discovered changes in histone occupancy for hotspot versus basal control, and the meiotic recombination phenotypes of null mutants lacking histone chaperones (described below), support a model in which the exchange of individual histone subunits contributes to hotspot activation.

### Multiple, combinatorial histone PTMs

We searched our histone peptide datasets for well-characterized histone PTMs: acetylation (ac), ubiquitination (ub), phosphorylation (ph), and mono-, di-, and tri-methylation (me1, me2, me3). This revealed changes in the relative abundance and temporal dynamics of histone PTMs in hotspot versus basal control MiniCs (Fig. 4b). For example, the H3K14ac mark was enriched transiently at the hotspot in meiosis (from 1 to 3 h, peaking at 2 h), which is consistent with findings made previously using ChIP of the chromosomal hotspot [17, 20]. In addition, we found that a histone acetyltransferase that writes this mark, Mst2 [59], binds to the hotspot (Fig. 3d) just before the time when maximum acetylation occurs (Fig. 4b). These findings are congruent with the fact that Mst2 is required for both chromatin remodeling and for high-frequency recombination at the hotspot [57]. We conclude that our MiniC-AP-MS approach can identify hotspot-enriched histone PTMs that are *bona fide* regulators of recombination hotspots in chromosomes.

We also uncovered a constellation of additional histone PTMs that were enriched or depleted in the hotspot MiniC, relative to basal control, at one or more of the time points of meiosis (Fig. 4b) but that were not implicated previously to regulate meiotic recombination. These marks were also dynamic, suggesting that they, like the known regulator H3K14ac and like the regulatory proteins described above (e.g., Fig. 3d), are transient intermediates in meiotic chromatin of the hotspot. Remarkably, in the majority of the instances that we detected a peptide with a PTM, that peptide was multiply modified (34 different combinations of PTMs) (Fig. 4b). Additional, potentially regulatory proteoforms might be discovered in our bLC-LC–MS/MS datasets (which are available to the public) because the analyses described here were restricted to a limited subset of well-defined histone PTMs (ac, ub, ph, me1, me2, and me3). Such combinatorial modifications within individual histone molecules, which are undetectable by ChIP [60–62], might function together to help position meiotic recombination (see Discussion).

### Newly discovered, hotspot-enriched factors are *bona fide* regulators of hotspot activation

To test whether factors that were enriched within the hotspot MiniC actually regulate meiotic recombination, we selected nine different proteins with known or putative roles in chromatin remodeling. Each of these proteins was enriched at the hotspot, relative to basal control, at one or more time points of meiosis in at least one experiment, or was a component of a well-defined protein complex with multiple subunits enriched at the hotspot, or both (Additional file 1: Table S2 and ProteomeXchange datasets). Our choice of which proteins to analyze was also guided by genetic constraints. For example, we detected multiple subunits of the Ino80 complex, but because the gene encoding the catalytic subunit (*ino80*) is essential, we chose to analyze two genes encoding two non-essential subunits of the complex (*arp5* and *arp8*). Our selection of candidates was also guided by the proteomics results on histone occupancy and PTMs, as follows.

Because we observed changes over time in the relative occupancy of individual histones at the hotspot (Fig. 4a), we selected histone chaperones known to mediate the exchange of histone subunits within nucleosomes. These included the H2A-H2B chaperone Nap1 [63–65], the H3-H4 chaperone Hip1 (Hir1) [66–70], plus Arp5 and Arp8, which are non-essential subunits of the multifunctional Ino80 chromatin remodeling complex whose activities include the eviction of H2A.Z [71, 72]. Because hotspot activation involves nucleosome displacement [17], we also chose to analyze the Swr1 and Swc2 subunits of the Swr1 complex, which is a Swi2/Snf2 family ATP-dependent DNA helicase that remodels chromatin structure [73, 74]. Similarly, we selected the Fft3 (Fun30) protein because it helps to evict nucleosomes during the repair of DSBs in mitotic cells [75, 76]. Lastly, to test potential modifying enzymes, we selected the DNA helicase/E3 ubiquitin ligase Rrp2 [77, 78] and a putative protein phosphatase encoded by *spbc16h5.12* [79].

**Fig. 5:**
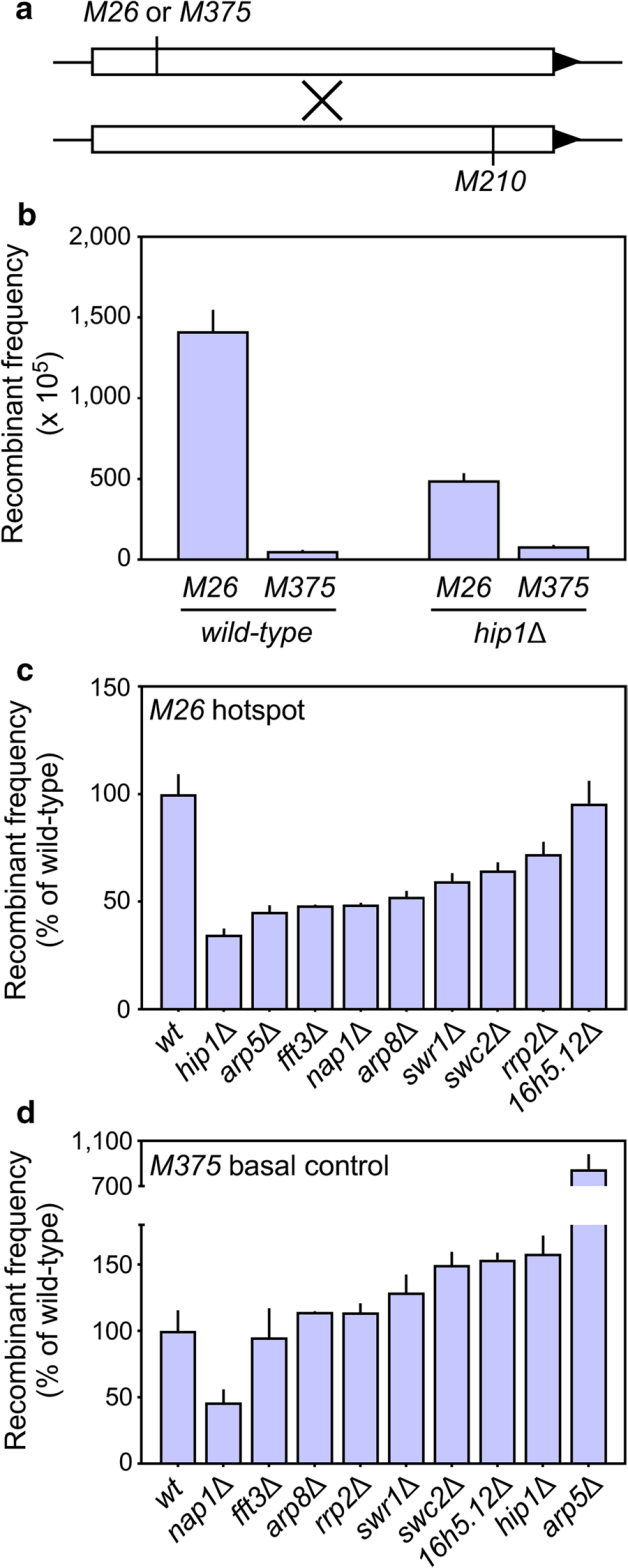
Newly discovered, hotspot-enriched proteins are *bona fide* regulators of hotspot activation. **a** The frequencies of meiotic recombination were determined in test crosses harboring hotspot (*M26*) and basal control (*M375*) alleles of *ade6* in the chromosome. **b** Example of a hotspot-specific regulator, plotted using recombinant frequencies. Note that the removal of the Hip1 (Hir1) protein reduces recombination at *M26* (hotspot) but not at *M375* (basal recombination control), indicating that the basal recombination machinery is intact. **c** The frequencies of hotspot recombination at *M26* were determined in strains with the indicated genotypes and are plotted as percent activity relative to that in wild-type cells. **d** As in “c,” but showing the effects of the null mutations on basal recombination at *M375.* Data are mean ± SD from three or more biological replicates

To see whether these enzymes regulate recombination in the genome, we constructed strains that were null mutant for the respective proteins and that contained additional markers with which to measure recombination (Additional file 1: Table S1). Haploid strains with hotspot (*M26*) and basal control (*M375*) alleles of *ade6* were crossed to a tester strain (*M210*) and spore colonies were genotyped to determine the frequency of Ade + recombinants (Fig. 5a). In wild-type cells, the recombinant frequency for *M26* was much higher than that for *M375*, demonstrating that the hotspot is active (Fig. 5b). In cells lacking Hip1 (Hir1), there was no significant reduction in recombination for *M375*, demonstrating that the basal recombination machinery is intact (i.e., the Rec12/Spo11 complex and other general recombination factors are expressed and functional). However, the ablation of Hip1 reduced substantially the frequency of recombination at *M26* (Fig. 5b). We conclude that the histone H3-H4 chaperone Hip1, which is recruited directly or indirectly to the *M26* hotspot by the Atf1-Pcr1-*M26* protein–DNA complex (Fig. 3d), is a *bona fide* regulator of hotspot activation that helps to position the local activity of the basal recombination machinery.

Analyses of the other candidates were equally informative. Eight of the nine deletion mutations reduced significantly the frequency of meiotic recombination at the *M26* hotspot (Fig. 5c). For seven of these, there was no significant reduction in basal recombination at *M375* (Fig. 5d), demonstrating that the encoded proteins (Arp5, Arp8, Fft3, Hip1, Rrp2, Swr1 and Swc2) contribute specifically to hotspot activation. The removal of the Nap1 protein attenuated significantly recombination for both *M26* and *M375*, so this protein also contributes to hotspot activation. However, our current data cannot distinguish whether it affects the positioning of the basal recombination machinery, or its overall catalytic potential, or both. Surprisingly, while Arp5 was required for high-frequency recombination at the *M26* hotspot, its removal increased substantially (nearly tenfold) recombination at *M375*. We obtained the same result using additional hotspot and control alleles (our unpublished observations). Therefore, Arp5 exhibits dual specificity as a repressor of basal recombination and as an activator of hotspot recombination. These phenotypes are, in each case, attributable presumptively to the local binding of Arp5 because it was enriched at the hotspot early in meiosis (1 h) and at the basal control later in meiosis (3 h) (Additional file 1: Table S2). Three other proteins (Swc2, Spbc16h5.12 and Hip1) also repressed basal recombination (Fig. 5d), although their effects were modest.

## Discussion

### Strengths and limitations of the approach

The MiniC-AP-MS approach allowed us to purify to near homogeneity, under native conditions (without chemical cross-linking), discrete, small segments of chromatin (4.2 kbp) bearing a meiotic recombination hotspot (*M26*) or a basal recombination control (*BC*) that differ by only two base pairs (Figs. 1, 2). With this approach, we defined the abundance of associated proteins and histone PTMs at sequential time points of meiosis preceding and up to the point when Rec12 (Spo11) first catalyzes the formation of recombination-initiating DSBs (Figs. 2, 3, 4 and ProteomeXchange datasets) [52].

Our approach was, in essence, a technologically sophisticated screen for candidate hotspot regulators by virtue of association. As with all screens (e.g., classical genetics, synthetic lethality, two-hybrid, drug sensitivity), it is not without limitations. There are likely false negatives due to the dissociation of labile proteins during purification under the native, stringent conditions employed. In addition, some proteins and (in particular) histone PTMs that are present can escape detection due to paucity of tryptic peptides that fall within the mass-to-charge gates of MS instrumentation. Other histone PTMs were missed simply because we focused on well-characterized PTMs (ac, ub, ph, me1, me2, me3). Components of the basal recombination machinery, such as Rec12, were also not detected at meaningful levels in our samples, which is not surprising because DSBs occur in only about 1% of DNA molecules harboring *ade6*-*M26* [52]. In addition to false negatives, false positives can occur due to the artifactual association, ex vivo, of proteins in homogenized extracts with components of the hotspot MiniC. Thus, as with all screens used for discovery, the utility of our approach must be validated by answering two key questions. First, does the MiniC-AP-MS screen for recombination hotspot-associated factors detect ones that are already known to regulate hotspot activity? Second, are newly implicated factors required for biological activity in vivo? The answer, in each case, is yes.

### New insight into pathway mechanisms of known hotspot regulators

The first criterion, on the screen’s ability to identify known regulators, was satisfied at the level of sequence-specific DNA binding proteins, histone- and chromatin-modifying enzymes, histone PTMs, and changes in histone/nucleosome occupancy.

Binding of the Atf1-Pcr1 (Mts1–Mts2) heterodimer [25] to the *M26* DNA site is essential for hotspot activity at *ade6*-*M26* and other *M26* DNA sites in the genome [5, 12, 25, 80], and our approach revealed that each subunit of the heterodimer was recruited to the hotspot (Fig. 3d). Moreover, Atf1 and Pcr1 were each enriched at the same time points, as expected for subunits of the same complex. Thus, the MiniC-AP-MS screen detected successfully known components of the primary regulatory module.

The previously reported, Atf1-Pcr1-*M26* protein–DNA complex-dependent, meiotically induced, transient accumulation (as determined by ChIP) of histone H3K14 acetylation at the hotspot [17, 20] was also recapitulated by our MiniC-AP-MS analyses of hotspot versus basal control (Fig. 4b). Thus, the screen can successfully identify hotspot-dependent histone PTMs and dynamic changes in their relative abundance during progression through meiosis.

A histone acetyltransferase that writes the H3K14ac mark, Mst2 [59], and an ATP-dependent chromatin remodeling factor, Snf22, each contribute to hotspot-specific chromatin remodeling and to hotspot recombination [17, 57]. However, neither protein had been localized to the hotspot. Our MiniC-AP-MS approach revealed that these enzymes are each recruited to the hotspot (Fig. 3d), providing reciprocal confirmation of the main findings in each study and completing the chain of evidence: These enzymes are each at the right place at the right time to mediate directly, in *cis*, changes in chromatin structure during activation of the hotspot.

Recently, ChIP analyses of a single meiotic time point revealed that the occupancy of the histone H2A variant H2A.Z is low at the *M26* hotspot [38]. This finding, too, was recapitulated by our MiniC-AP-MS analyses (Fig. 4a), providing further evidence of the screen’s utility. In addition, our time course analyses revealed that there are temporally phased changes in the relative abundance of each histone within the MiniCs (Fig. 4a). These changes, and the phenotypes of mutants lacking two different histone chaperones and subunits of an H2A.Z-evicting protein complex (Fig. 5), provide new insight into chromatin-based mechanisms of hotspot activation (discussed subsequently).

At each of the levels described above, findings made using MiniC-AP-MS (Figs. 3; 4) are concordant with findings made previously using orthogonal methods (e.g., ChIP, nuclease sensitivity assays and phenotyping of mutants). Moreover, because our approach revealed simultaneously the abundance of multiple factors within individual samples, across sequential time points of meiosis, it provides new information about the potential order of function of known regulatory factors. For example, the hotspot-activating Atf1-Pcr1 heterodimer and the histone H3K14 acetyltransferase Mst2 were recruited early (at the 1-h time point), followed subsequently (at 2 h) by the acetylation of histone H3K14 and the recruitment of the Snf22 chromatin remodeling enzyme (Figs. 3, 4). In summary, our MiniC-AP-MS screen for hotspot-regulating factors works, providing additional information on known and candidate regulators, molecular mechanisms and potential order of function within pathways.

### Many additional chromatin remodeling factors regulate hotspot activity

The second criterion, on whether newly discovered factors are required for biological activity in vivo, was satisfied by comparing rates of meiotic recombination in the genomes of wild-type cells and null mutants (Fig. 5).

Eight different chromatin remodeling factors—including histone H2A-H2B and H3-H4 chaperones (Nap1, Hip1) that mediate the exchange of individual histones within nucleosomes [63–70], subunits of the multifunctional Ino80 complex (Arp5, Arp8) whose activities include the eviction of H2A.Z [71, 72], a DNA helicase/E3 ubiquitin ligase (Rrp2) [77, 78], components of a Swi2/Snf2 family remodeling complex (Swr1, Swc2) [73, 74] and a nucleosome evictor (Fft3) [75, 76]—were each required for full hotspot activity at chromosomal *ade6*-*M26* (Fig. 5). For seven of these enzymes (all except for Nap1), the removal of the respective protein caused no significant reduction in basal meiotic recombination at *ade6*-*M375*, demonstrating that all components of the basal recombination machinery are intact and functional. Thus, these chromatin remodeling factors each regulate the positioning of recombination at hotspots. Interestingly, Arp5 binds preferentially (but at different time points) to both the hotspot and basal control (Additional file 1: Table S2) and it exhibits dual specificity in that it is required for promoting recombination at the *M26* hotspot and for repressing recombination at the *M375* basal control (Fig. 5c, d). This finding, too, is consistent with local chromatin structure and dynamics regulating where the basal recombination machinery acts along chromosomes. Numerous additional, hotspot-enriched proteins (Fig. 3d, Additional file 1: Table S2 and publically available datasets) remain to be tested for their roles in recombination.

Our findings (proteomics-based discovery and the documentation of biological activities in vivo) increase substantially the number of chromatin remodeling factors known to help position meiotic recombination at hotspots, and they provide new mechanistic insight. The requirement for histone chaperones (Nap1, Hip1, and Ino80C subunits Arp5 and Arp8) (Fig. 5c), coupled with temporally phased changes in the relative occupancy of histones (Fig. 4a), provide strong evidence that histone subunit exchanges are intermediates in hotspot activation. Another intermediate, exemplified by the need for ATP-dependent chromatin remodeling enzymes and nucleosome evictors (Fft3, Swr1, and Swc1), likely involves the displacement of entire nucleosomes (by sliding, eviction or both). And the DNA helicase/E3 ubiquitin ligase (Rrp2) might regulate posttranslationally other chromatin remodeling factors or recombination proteins (via ubiquitination), or participate more directly in chromatin remodeling (via its helicase activity), or both.

### Potentially regulatory histone codes

Our analyses also uncovered a constellation of differentially abundant histone PTMs (Fig. 4b), including one (H3K14ac) already known to be enriched at the hotspot [17, 20] and whose acetyltransferase (Mst2) contributes to hotspot activation [57]. By extension, the additional, hotspot-enriched, individual and combinatorial PTM marks on histones are potential activators, whereas the marks that are enriched at the basal control are potential repressors. The timing of their appearance is likely important. As reported previously [17] and confirmed in this study (Fig. 4b), hotspot-enriched histone PTMs implicated to help position recombination can decline in abundance before the time when the basal recombination machinery catalyzes the formation of DSBs, suggesting that they are transient intermediates of the pathway(s). Such intermediates could function through the sequential actions of known (and unknown) hotspot-regulating proteins, such as Gcn5 [17], which is both a histone code reader (via its bromodomain) and writer (via its acetyltransferase domain). We therefore suggest that combinatorial histone PTMs and their order of function confer additional specificity to where the basal recombination machinery acts. Additional, emerging data support this hypothesis. For example, at Prdm9-dependent hotspots the simultaneous presence of H3K4me3 and H3K36me3 is a better predictor of hotspot activity than each mark alone [81]. Looking forward, it will be interesting to test whether individual and combinatorial PTMs discovered in this study (Fig. 4b) are *bona fide* regulators of hotspot activity.

## Conclusions

Overall, our findings indicate that a remarkably diverse collection of chromatin remodeling factors and (hypothetically) histone modifications participate in designating where meiotic recombination occurs in the fission yeast genome. To the extent tested, these factors also regulate other DNA sequence-dependent hotspots of *S. pombe* (our unpublished observations). Fundamental aspects of hotspot control in fission yeast—including its regulation in *trans* by signal transduction pathways [26, 36, 82, 83] and in *cis* [84] by DNA sequence-specific binding proteins [12–14, 25], histone PTMs and chromatin remodeling enzymes [17, 20, 37, 57]—are employed by diverse organisms (see Introduction). Notably, each of the chromatin-mediated regulators of hotspot activity discovered in this study (Arp5, Arp8, Fft3, Hip1, Nap1, Rrp2, Swr1 and Swc2) (Fig. 5) is conserved broadly across eukaryotic taxa (Table 1), suggesting that they might help to position the catalytic activity of the basal recombination machinery in diverse taxa. Lastly, the use of multiple different chromatin remodeling factors (Fig. 5) and the inferred use of combinatorial histone PTMs (Fig. 4) to help position recombination provides an explanation for why individual factors and PTMs that are required for hotspot activation display context variable penetrance (*i.e*., they are insufficient to promote recombination at many of their locations in the genome). Multiple epigenetic and chromatin factors function in concert (together or sequentially) to position meiotic recombination at hotspots.

**Table 1:** Chromatin remodeling factors that regulate meiotic recombination are broadly conserved

Fission yeast	Budding yeast	Thale cress	Worm	Fly	Zebrafish	Frog	Rat	Mouse	Human
*Arp5*	*ARP5*	*ARP5*	–	*Arp5*	*actr5*	*actr5*	*Actr5*	*Actr5*	*ACTR5*
*Arp8*	*ARP8*	*ARP9*	–	*Arp8*	*actr8*	*actr8*	*Actr8*	*Actr8*	*ACTR8*
*Fft3*	*FUN30*	*ETL1*	*M03C11.8*	*Etl1*	*smarcad1a*	*smarcad1*	*Smarcad1*	*Smarcad1*	*SMARCAD1*
*Hip1*	*HIR1*	*HIRA*	*hira*-*1*	*Hira*	*hira*	*hira*	*LOC100911837* *Hira*	*Hira*	*HIRA*
*Nap1*	*NAP1*	*NAP1;1* *NAP1;2* *NAP1;3*	*nap*-*1*	*Nap1*	*nap1l1*	*nap1l1*	*Nap1l1*	*Nap1l1*	*NAP1L1*
*Rrp2*	*ULS1*	*AT1G50410* *AT3G20010* *EDA16*	*T23H2.3* *F54E12.2*	*lds*	*hltf*	–	*Hltf*	*Hltf*	*HLTF*
*Swc2*	*VPS72*	*SWC2*	*C17E4.6*	*YL*-*1*	*vps72*	–	*Vps72*	*Vps72*	*VPS72*
*Swr1*	*SWR1*	*PIE1*	*ssl*-*1*	*dom*	*srcap*	*ep400*	*Srcap*	*Ep400*	*SRCAP*

Names of the fission yeast genes are provided along with their best predicted orthologs in other species as identified using the DRSC Integrative Ortholog Prediction Tool. Blank cells (–) indicate no “best” (i.e., top-scoring) ortholog, even though orthologs might be present

## Methods

### Molecular biology

Standard recombinant DNA methods were used to construct minichromosomes (MiniCs) with components shown in Fig. 2a. Initial constructions were made in the *E. coli* plasmid pBluescript II KS- and, after DNA sequencing to confirm the correct structure and to eliminate any clones with spurious mutations, that plasmid was digested with *Not*I to liberate the *S. pombe* sequences. The linear MiniC DNA fragment was isolated by gel electrophoresis, was ligated at very low DNA concentration (1 ng/ml) to favor intramolecular ligation (circularization) over intermolecular ligation (concatemerization), and was transformed into *S. pombe* [48]. The unit structure of MiniCs within fission yeast was confirmed by a combination of diagnostic PCR and DNA sequencing.

Quantitative, real-time PCR (All-in-One qPCR Master Mix, GeneCopoeia) was used to measure MiniC copy number and degree of enrichment during purifications. The relative abundance of *ade6* DNA in the chromosome, the MiniCs, and in MiniCs from steps of affinity purification was normalized to a single-copy genomic control, *act1*, and relative abundance values were calculated using the double-delta Ct method [85]. The sequences of primers used to amplify *ade6* were: 5′-CAATTGGGCCGAATGATGGT-3′ and 5′-TTTCGTAACGGCTGCCAAGG-3′; those for *act1* were 5′-GAAATCGCAGCGTTGGTTAT-3′ and 5′-ACGCTTGCTTTGAGCTTCAT-3′.

### Genetic methods and meiotic inductions

Genotypes of *S. pombe* strains used in this study are provided in Additional file 1: Table S1. These were constructed using standard genetic techniques and were cultured on rich media or minimal media supplemented as appropriate with amino acids and bases at 100 µg/ml and G418 at 100 µg/ml [86]. Minichromosomes (MiniCs) were maintained by selection for adenine prototrophy in an *ade6*-*D1 sup35*-*F592S* strain background, which suppresses the *ade6* mutations in the MiniCs [48]. Recombinant frequencies were determined by genotyping spore colonies from genetic crosses [12, 26].

Meiosis was induced by thermal inactivation of the Pat1-114^ts^ repressor, as described [50], but with the following modifications. We used *pombe* minimal media [86] containing 1% glucose and 3 g/L of glutamate as the nitrogen source (PMG). Cultures were grown at 25 °C, splitting as necessary, and cells were synchronized in G0 (G1) phase of the cell cycle by incubating for 16 h once they had reached the inflection point between log phase and stationary phase. Cultures were diluted 1:4 into fresh PMG, incubated at 25 °C for 1 h to permit recovery from starvation, and then brought rapidly to 34 °C (by swirling the flasks in a hot water bath) to induce meiosis. Flasks were returned to a 34 °C incubator, and samples were collected at hourly time points. Cells were harvested by centrifugation (1800×*g* for 5 min at 4 °C), washed once with ice-cold ddH_2_O, collected by centrifugation, resuspended in the residual liquid, and snap-frozen by drizzling into liquid N_2_. The frozen cell “popcorn” was stored at − 80 °C until processed further.

To monitor meiotic progression, cells (1 ml of culture) were taken at hourly time points, fixed with 70% ethanol, and stored at − 20 °C. Following rehydration with sodium citrate, cells were spread onto microscope slides, dried with heat, and then stained with 1 μg/ml of 4,6-diamidino-2-phenylin-dole (DAPI) and antifade solution (0.25% DABCO in 75% glycerol/25% PBS). Cells were visualized and analyzed with the EVOS FL Auto Imaging System using phase contrast and fluorescence microscopy. A minimum of 100 cells were counted for each sample at each time point.

### Affinity purification of minichromosomes

Frozen cells were lysed under cryogenic conditions using a Retsch MM301 ball mill, at power setting of 30 Hz, with 5 cycles of 3 min each (at each cycle, the steel cylinder was re-cooled in liquid N_2_). The resulting frozen cell lysate powder was stored at − 80 °C until processed further. For each affinity purification, 6 g of frozen cell lysate powder was thawed on ice; all subsequent steps were carried out on ice or at 4 °C. To the 6 g (approximately 6 ml) of thawed lysate, we added 25 ml of buffer A150 (25 mM HEPES–KOH pH 7.6, 150 mM KCl, 2 mM MgCl_2_, 1 mM EDTA, 0.5 mM EGTA, 10% glycerol, 0.02% NP40). The A150 buffer was supplemented with protease inhibitors (to 1 mM PMSF, 0.5 µg/ml leupeptin, 0.7 µg/ml pepstatin, 1 µg/ml aprotinin), phosphatase inhibitors (2 mM imidazole, 1 mM sodium fluoride, 1.15 mM sodium molybdate, 1 mM sodium orthovanadate), deacetylase inhibitors (500 µM butyric acid), 0.5% (v/v) Triton X-100, 125 µM spermidine, and 50 µM spermine. The solution was centrifuged at 2600×*g* for 5 min, and the supernatant was collected; then, that material was centrifuged at 12,000×*g* for 30 min. The supernatant was collected as clarified whole-cell extract (WCE), 20 µL of aliquots was saved (to measure amounts of protein and DNA), and the remaining WCE was used for affinity purification. The WCE was pre-cleared with 500 µL of IgG-Sepharose beads (GE Lifesciences), equilibrated in A150 buffer, for 15 min on a rotator at 4 °C. The Sepharose beads were removed by two rounds of centrifugation, each for 5 min at 2600×*g*. Six hundred ng of LacI-6xhis-prA fusion protein was added to the WCE, which was then incubated on a rotator at 4 °C for 15 min. Twelve milligrams of Rabbit IgG-conjugated magnetic Dynabeads (Thermo Fisher) was added, and the sample was incubated for 2 h on a rotator at 4 °C. The Dynabeads (and adsorbed material) were first collected by centrifugation for 5 min at 2600×*g*. All but 1 ml of supernatant was removed, and the Dynabeads were resuspended in the remaining liquid. That material was then split into 3 separate 1.7-ml Eppendorf tubes, the magnetic beads were collected using a magnetic rack, and the supernatant was removed. The same process was used to wash the beads sequentially 3 times, for 5 min each, with 1 ml of A300 buffer (like A150 described above, but with 300 mM KCl) per tube. For the final wash, the beads were transferred to new 1.7-ml Eppendorf tubes. A small aliquot of beads (5% v/v in wash buffer) was collected to test for enrichment of target DNA. The remaining beads (95% v/v) were pooled into one tube, recovered magnetically, resuspended in 30 µL of 1 × SDS-PAGE loading buffer, brought to 100 µL volume with ddH_2_O, and incubated at 95 °C for 10 min. The eluant was separated from the Dynabeads by centrifugation, collected, and transferred to a new Eppendorf tube. This process was repeated one more time to ensure that no beads were collected. The samples were dried overnight in a DNA 120 SpeedVac concentrator (Thermo Fisher) at room temperature.

### Mass spectrometry

Purified proteins were reduced, alkylated, and digested using filter-aided sample preparation [87]. Tryptic peptides were separated into 36 fractions on a 100 × 1.0 mm Acquity BEH C18 column (Waters) using an UltiMate 3000 UHPLC system (Thermo) with a 40-min gradient from 99:1 to 60:40 buffer A:B ratio under basic pH conditions (buffer A is 0.05% acetonitrile with 10 mM NH_4_OH; buffer B is acetonitrile with 10 mM NH_4_OH). The individual fractions were then consolidated into 12, 18, or 24 super-fractions, each of which was then further fractionated by reverse phase chromatography on a Jupiter Proteo resin (Phenomenex) on an in-line 150 × 0.075 mm column using a nanoAcquity UPLC system (Waters). Peptides were eluted using a 60-min gradient from 97:3 to 65:35 buffer A:B ratio (buffer A is 0.1% formic acid; buffer B is acetonitrile plus 0.1% formic acid). Eluted peptides were ionized by electrospray (2.15 kV) followed by MS/MS analysis using higher-energy collisional dissociation (HCD) on an Orbitrap Fusion Tribrid mass spectrometer (Thermo) in top-speed data-dependent mode. MS data were acquired using the FTMS analyzer in profile mode at a resolution of 240,000 over a range of 375–1500 m/z. Following HCD activation, MS/MS data were acquired using the ion trap analyzer in centroid mode and normal mass range with precursor mass-dependent normalized collision energy between 28.0 and 31.0. Proteins were identified by database search using MaxQuant (Max Planck Institute) with a parent ion tolerance of 3 ppm and a fragment ion tolerance of 0.5 Da. Carbamidomethylation of cysteine residues was used as a fixed modification. Acetylation of protein N-termini and oxidation of methionine were selected as variable modifications. Protein abundance was calculated using the intensity-based absolute quantification (iBAQ) algorithm [53–55]. Histone PTMs were detected by Mascot search engine (Matrix Science). The relative abundance of histone PTMs at a given site was determined as abundance of the modified peptide relative to abundance of all peptides (modified and unmodified) that span the modified site. This approach controls for changes in protein abundance and provides a measure of changes in PTM stoichiometry independent of changes in protein abundance. Data normalization and analyses were performed using R.

## Additional file

**Additional file 1.** Contains supplementary information germane to but not essential for inclusion in the main text: Fig. S1, purification and DNA binding of LacI-6xHis-PrA fusion protein; Fig. S2, optimization of conditions for purification of MiniCs; Fig. S3, normalization of MS data for differences in protein yield; Fig. S4, Pearson correlation coefficients for all pairwise combinations; Table S1, genotypes of *S. pombe* strains used; Table S2, nuclear and chromatin-associated proteins enriched at the hotspot.

## Abbreviations

ac: acetylation
AP: affinity purification
ARS: autonomously replicating sequence
BC: basal control
bLC: basic pH liquid chromatography
ChAP-MS: chromatin affinity purification with mass spectrometry
ChIP: chromatin immunoprecipitation
Chr: chromosome
DSB: double-strand DNA breaks
iBAQ: intensity-based absolute quantification
IP: immunopurification
LC: liquid chromatography
me: methylation
MI: first meiotic division
MII: second meiotic division
MiniC: minichromosome
MiniC-AP-MS: minichromosome affinity purification with mass spectrometry
MS: mass spectrometry
NDR: nucleosome depleted region
ph: phosphorylation
PTM: posttranslational modification
qPCR: quantitative polymerase chain reaction
ub: ubiquitination
