# Supplementary material for: Chromatin-mediated regulators of meiotic recombination revealed by proteomics of a recombination hotspot
Source: Epigenetics Chromatin. 2018 Oct 29;11:64. doi: 10.1186/s13072-018-0233-x (PMC6205778; doi:10.1186/s13072-018-0233-x)
Supplement: Supplementary file 1 — Additional file 1. Contains supplementary information germane to but not essential for inclusion in the main text: Fig. S1, purification and DNA binding of LacI-6xHis-PrA fusion protein; Fig. S2, optimization of conditions for purification of MiniCs; Fig. S3, normalization of MS data for differences in protein yield; Fig. S4, Pearson correlation coefficients for all pairwise combinations; Table S1, genotypes of S. pombe strains used; Table S2, nuclear and chromatin-associated proteins enriched at the hotspot. [file 13072_2018_233_MOESM1_ESM.pdf]

Epigenetics & Chromatin

Additional file 1 (supplementary material)

Chromatin-mediated regulators of meiotic recombination revealed by proteomics of a recombination hotspot

Aaron J. Storey, Hsin-Ping Wang, Reine U. Protacio, Mari K. Davidson, Alan J. Tackett, and Wayne P. Wahls*

This file contains:

Figure S1. Purification and DNA binding of LacI-6xHis-prA fusion protein
Figure S2. Optimization of conditions for purification of MiniCs
Figure S3. Normalization of MS data for differences in protein yield
Figure S4. Pearson correlation coefficients for all pairwise combinations
Table S1. Genotypes of *S. pombe* strains used in the study
Table S2. Nuclear and chromatin-associated proteins enriched at the hotspot

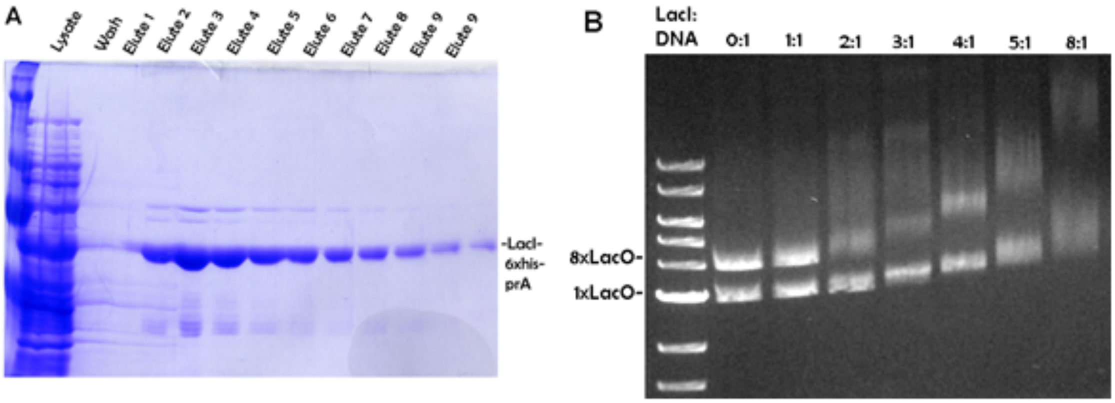

Figure S1. Purification and DNA binding of LacI-6xHis-prA fusion protein. (**A**) Fusion protein expressed in *E. coli* was purified by metal-affinity chromatography; SDS-PAGE shows sequential fractions from elution. (**B**) Plasmid DNA molecules bearing 1 and 8 copies of the *LacO* DNA site were incubated with the indicated amounts (stoichiometry) of LacI-6xHis-prA; binding reactions were analyzed by agarose gel electrophoresis. The shift in electrophoretic mobility reveals that the LacI moiety still binds efficiently to *LacO* DNA when fused to 6xHis-prA. The progressive increase in shift with increasing protein concencentration is as expected given the multivalency of LacI-*LacO* protein-DNA interactions and the number of *LacO* DNA sites that are present. Two aspects of this fusion protein helped us to achieve the extraordinary level of purification of the MiniCs (Figure 2). The first is that interactions between the LacI repressor and the *LacO* DNA sites, and between prA and the Fc portion of IgG, each occur with high affinity. The second is high valency for each binding configuration: the prA tag contains 4 Fc-binding domains (4x Fc); LacI binds to *LacO* as a tetramer (4x LacI); each MiniC contains multiple copies of the binding site (8x *LacO*). Thus there are 128 different binding combinations, located in close proximity, which contribute cooperatively to the affinity and avidity of the binding reactions used to enrich the MiniCs.

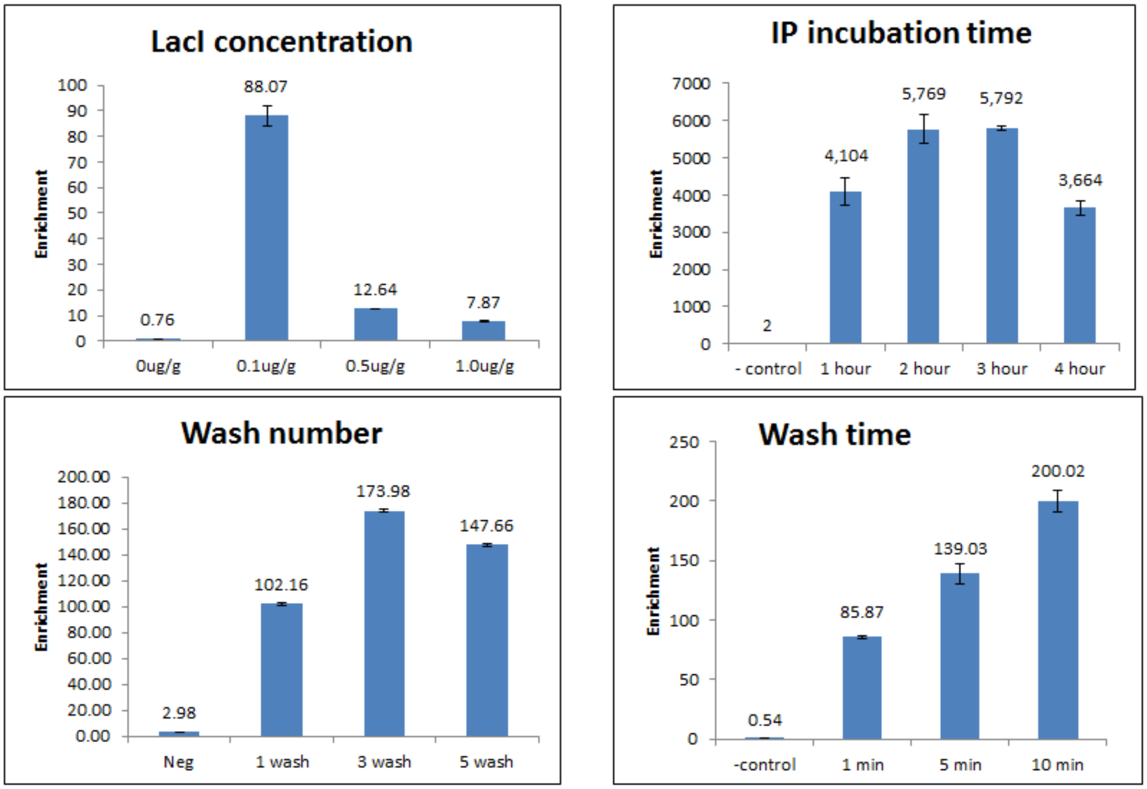

Figure S2. Optimization of conditions for purification of MiniCs. Affinity purification experiments were performed under similar conditions aside from the indicated variables. Detailed descriptions of the approach (buffers, order of addition, etc.) and for how we determined the degree of enrichment of MiniCs (using real-time qPCR) are provided in the main text. The most critical parameter was the ratio of LacI-6xhis-prA (the affinity capture ligand) to protein in the cell extract (top left). The degree of enrichment was also sensitive to how long the magnetic IgG-dynabeads (used to capture LacI-6xHis-prA and its MiniC cargo) were incubated with extract (top right). Following affinity capture, both the number (bottom left) and duration (bottom right) of washes also affected the degree of enrichment.

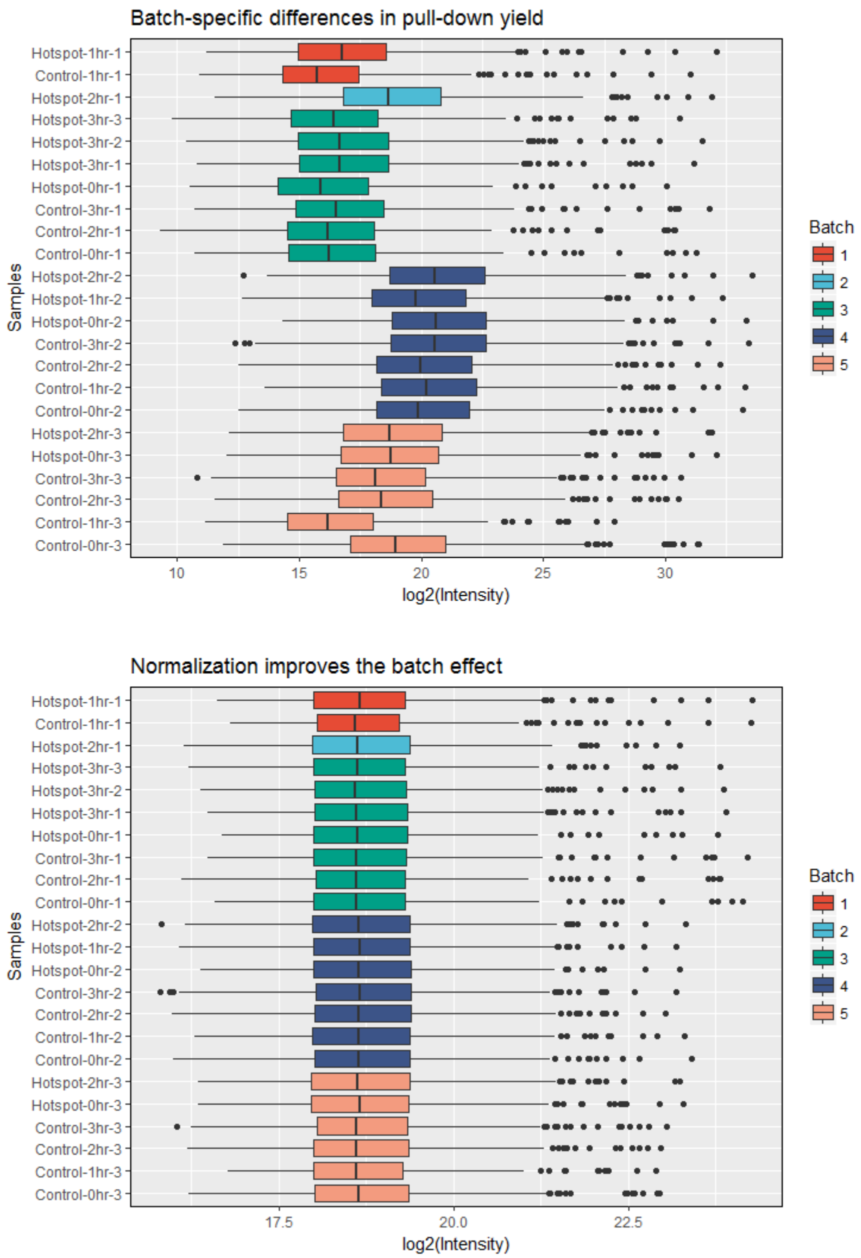

Figure S3. Normalization of MS data for differences in protein yield. Bar and whisker plots show the distribution of protein abundance (log2 iBAQ values) in each sample, with samples color-coded by batch in which each experiment was conducted. To adjust for the differences in protein yield between samples (top), the distributions within each sample were normalized to the median abundance across all samples. Note that after normalization the intensity distribution for each experiment and between batches are similar (bottom). Calculations were performed in R using the base scaling function and centered around the global median.

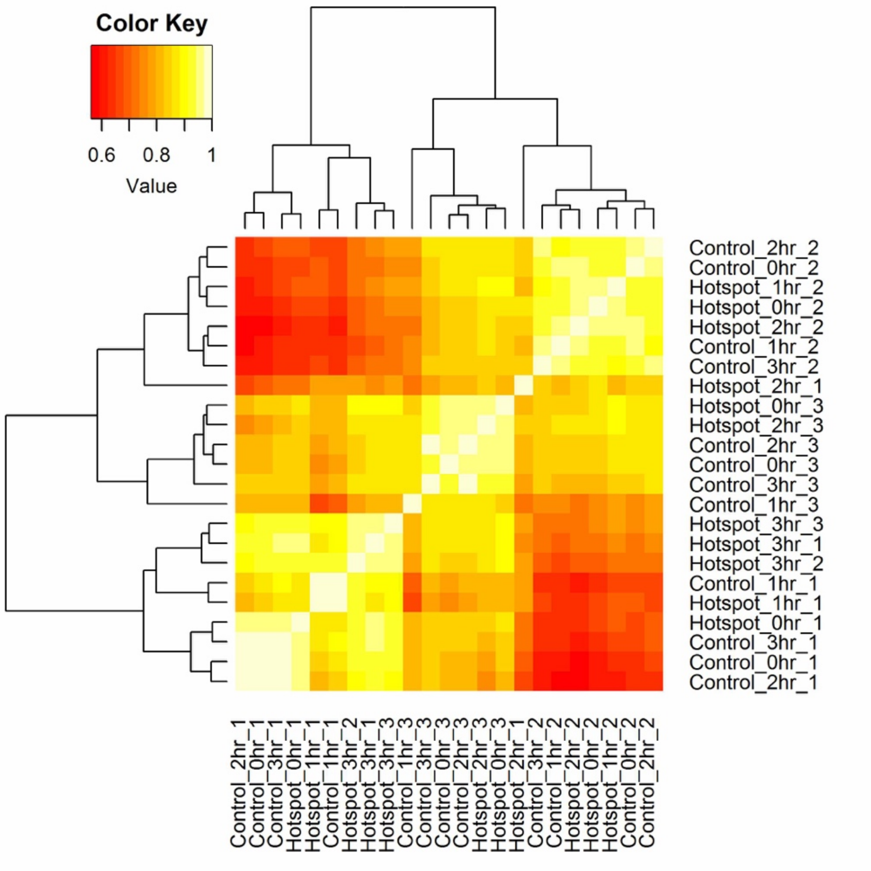

Figure S4. Pearson correlation coefficients for all pairwise combinations. As in **Figure 3a**, but with sample labels and dendograms applied. Heat maps show correlation coefficients (*r*) for all pairwise-combinations of protein data from all experimental conditions and all biological replicates, using the normalized data depicted in **Figure S3**. Hierarchical clustering was used to sort the data by *r* value; note that the "neighborhoods" correspond largely to experimental batches shown in **Figure S3**, indicating that batch effects persist to some extent even after normalization.

Table S1. Genotypes of *S. pombe* strains used in this study.

Strain	Genotype
WSP 5748	*h? lys3-37 arg1-230 car2D::kanMX4 pat1-114 ura4D18 leu1D::prALexA ade6-M26-LexABS*
WSP 5749	*h? lys3-37 arg1-230 car2D::kanMX4 pat1-114 ura4D18 leu1D::prALexA ade6-M375-LexABS*
WSP 5750	*h? ade6-D1 sup35-F592S pat1-114 ura4-D18 leu1D::prALacI (MiniC-M26, ade6-M26 8xLacO ARS1)*
WSP 5751	*h- ade6-M375 leu1-32 ura4-D18 (pREP41-NTAP-cdCas9)*
WSP 5752	*h- ade6-M375 leu1-32 ura4-D18 (pREP41-NTAP-cdCas9 pREP42-ade6-gRNA)*
WSP 5753	*h- ade6-M26 leu1-32 ura4-D18 (pREP41-NTAP-cdCas9)*
WSP 5754	*h- ade6-M26 leu1-32 ura4-D18 (pREP41-NTAP-cdCas9 pREP42-ade6-gRNA)*
WSP 5761	*h? ade6-D1 sup35-F592S pat1-114 (MiniC-M26, ade6-M26 8xLacO ARS1)*
WSP 5784	*h? ade6-D1 sup35-F592S pat1-114 (MiniC-BC, ade6-BC 8xLacO ARS1)*
WSP 6162	*h+ fft3D::kanMX4 ade6-M26*
WSP 6163	*h+ fft3D::kanMX4 ade6-M375*
WSP 6165	*h- fft3D::kanMX4ade6-M210*
WSP 6356	*h- SPBC16H5.12cD::kanMX4*
WSP 6402	*h- ade6-M210 swr1D::kanMX4*
WSP 6403	*h+ ade6-M210 swr1D::kanMX4*
WSP 6404	*h- ade6-M210 swc2D::kanMX4*
WSP 6405	*h+ ade6-M210 swc2D::kanMX4*
WSP 6410	*h+ ade6-M210 SPBC16H5.12cD::kanMX4*
WSP 6411	*h- ade6-M210 SPBC16H5.12cD::kanMX4*
WSP 6429	*h- ade6-M210 rrp2D::kanMX4*
WSP 6430	*h+ ade6-M210 rrp2D::kanMX4*
WSP 6431	*h- ade6-M210 hip1D::kanMX4*
WSP 6432	*h+ ade6-M210 hip1D::kanMX4*
WSP 6433	*h- ade6-M375 swr1D::kanMX4*
WSP 6434	*h- ade6-M375 swc2D::kanMX4*
WSP 6435	*h- ade6-M375 SPBC16H5.12cD::kanMX4*
WSP 6436	*h- ade6-M375 rrp2D::kanMX4*
WSP 6437	*h+ ade6-M375 hip1D::kanMX4*
WSP 6438	*h+ ade6-M26 swr1D::kanMX4*
WSP 6439	*h- ade6-M26 swc2D::kanMX4*
WSP 6440	*h+ ade6-M26 SPBC16H5.12cD::kanMX4*
WSP 6441	*h+ ade6-M26 rrp2D::kanMX4*
WSP 6442	*h- ade6-M26 hip1D::kanMX4*
WSP 6606	*h- arp8D::kanMX4 ade6-M26*
WSP 6608	*h- arp8D::kanMX4 ade6-M375*
WSP 6609	*h+ arp8D::kanMX4 ade6-M210 leu1-32*
WSP 6658	*h+ nap1D::kanMX4 ade6-M26*
WSP 6659	*h- nap1D::kanMX4 ade6-M375*

WSP 6660	*h+ nap1D::kanMX4 ade6-M375 leu1-32*
WSP 6661	*h+ nap1D::kanMX4 ade6-M210 leu1-32*
WSP 6662	*h- nap1D::kanMX4 ade6-M210*
WSP 6668	*h- arp5D::kanMX4 ade6-M26*
WSP 6669	*h- arp5D::kanMX4 ade6-M375*
WSP 6670	*h- arp5D::kanMX4 ade6-M210*
WSP 6671	*h+ arp5D::kanMX4 ade6-M210 leu1-32*

While standard nomenclature for fission yeast (and this table) uses a “D” to represent a gene deletion, such deletion mutations are referred to as “Δ” in the text and figures (e.g., *ade6-D1* is displayed as *ade6Δ*) to enhance clarity for those unfamiliar with fission yeast nomenclature. Extrachromosomal elements, such as the MiniCs, are in parentheses.

Table S2. Nuclear and chromatin-associated proteins enriched at the hotspot

			Log2 abundance (hotspot vs control)			
**Name**	**Description**	**Systematic id**	**0hr**	**1hr**	**2hr**	**3hr**
Ams2	CENP-A multicopy suppressor protein 2	SPCC290.04	0.00	0.00	4.76	0.00
Ap1	AP-1-like transcription factor	SPAC1783.07c	-0.59	6.10	-0.37	0.00
Arp4	Actin-related protein 4	SPBP23A10.08	-0.88	0.46	0.06	1.02
Arp5	Actin-like protein arp5	SPBC365.10	-0.64	1.06	-0.43	-4.62
Arp8	Probable actin-related protein 8	SPAC664.02c	0.00	5.10	0.00	5.95
Atf1	Transcription factor atf1	SPBC29B5.01	1.18	6.19	0.00	4.17
Bqt4	Bouquet formation protein 4	SPBC19C7.10	2.23	0.37	-0.70	0.05
Brl1	E3 ubiquitin-protein ligase brl1	SPCC1919.15	0.00	4.09	4.50	0.00
Brr6	Nucleus export protein brr6	SPAC8F11.06	0.39	5.90	0.00	-4.76
Bub1	Checkpoint serine/threonine-protein kinase bub1	SPCC1322.12c	-1.74	6.11	-2.90	0.37
Cbh2	CENP-B homolog protein 2	SPBC14F5.12c	3.54	0.00	0.05	1.32
Cgi121	EKC/KEOPS complex subunit cgi121	SPCC24B10.12	4.32	5.84	0.00	0.00
Csn1	COP9 signalosome complex subunit 1	SPBC215.03c	-0.07	0.34	4.87	-0.84
Csn2	COP9 signalosome complex subunit 2	SPAPB17E12.04c	-0.15	0.34	1.06	-4.05
Cyc8	General transcriptional corepressor ssn6	SPBC23E6.09	-0.21	0.07	1.89	0.70
Dbp5	ATP-dependent RNA helicase dbp5	SPBC12C2.06	1.04	-0.68	-0.25	-3.53
Dcp1	mRNA-decapping enzyme subunit 1	SPBC3B9.21	1.10	0.57	0.96	-4.97
Dcp2	mRNA decapping complex subunit 2	SPAC19A8.12	-1.42	4.12	3.08	-3.95
Dnli1	DNA ligase 1	SPAC20G8.01	1.33	0.79	0.67	-4.32
Dpb2	DNA polymerase epsilon subunit B	SPBP8B7.14c	4.11	-3.98	1.27	0.00
Dyl1	Dynein light chain 1, cytoplasmic	SPAC926.07c	4.90	0.04	2.22	-5.01
Ekc1	Extragenic suppressor of kinetochore protein 1	SPCC663.01c	-0.82	0.97	1.72	1.04
Ely5	Uncharacterized protein ely5	SPBC29A10.06c	0.91	0.95	1.39	-4.23
Far11	Factor arrest protein 11	SPBC27B12.04c	3.51	0.36	0.19	-4.29
Fft3	ATP-dependent helicase fft3	SPAC25A8.01c	-0.01	1.20	-0.44	-5.35
Fin1	G2-specific protein kinase fin1	SPAC19E9.02	0.00	0.00	4.46	-4.22
Flp1	Tyrosine-protein phosphatase CDC14 homolog	SPAC1782.09c	1.13	-3.90	4.01	-0.66
Gad8	Serine/threonine-protein kinase gad8	SPCC24B10.07	1.45	0.00	0.36	0.00

Glrx3	Monothiol glutaredoxin-3	SPCC1450.06c	0.20	5.18	1.54	4.08
Grc3	Polynucleotide 5-hydroxyl-kinase grc3	SPCC830.03	-3.58	5.61	3.43	0.00
Hir1	Protein hir1	SPBC31F10.13c	0.90	5.85	3.45	0.02
Hob1	Protein hob1	SPBC21D10.12	0.23	-0.28	2.39	-1.07
Hrp1	Chromodomain helicase hrp1	SPAC1783.05	-0.35	4.71	3.91	0.16
Hrp3	Chromodomain helicase hrp3	SPAC3G6.01	-4.53	0.00	1.20	0.45
Hsf	Heat shock factor protein	SPAC2E12.02	5.02	5.40	0.00	0.00
Hst2	NAD-dependent protein deacetylase hst2	SPCC132.02	1.10	0.29	1.26	0.09
Hus1	Checkpoint protein hus1	SPAC20G4.04c	6.54	-9.62	2.00	0.00
Iec1	INO80 complex subunit 1	SPAC144.02	4.77	5.24	-0.08	-5.79
Iec5	INO80 complex subunit 5	SPAPB1E7.14	-4.47	5.91	0.00	0.00
Ies2	INO80 complex subunit 2	SPAC6B12.05c	-0.89	1.81	-1.11	0.00
Ies6	Chromatin-remodeling complex subunit ies6	SPAC222.04c	-5.24	5.52	-3.88	-4.68
Ima2	Importin subunit alpha-2	SPBC1604.08c	0.00	0.00	1.29	-4.55
Imb3	Importin subunit beta-3	SPCC1840.03	0.63	0.28	1.45	-4.00
Imp3	U3 small nucleolar ribonucleoprotein protein imp3	SPAC19D5.05c	0.42	-0.09	0.24	5.14
Kms1	Karyogamy meiotic segregation protein 1	SPAC3A11.05c	3.08	3.17	-0.13	0.00
Kms2	Karyogamy meiotic segregation protein 2	SPBC947.12	3.87	4.69	1.08	-4.81
Lem2	Lap-Emerin-Man domain protein 2	SPAC18G6.10	3.73	0.26	-2.07	-4.57
Lsm8	U6 snRNA-associated Sm-like protein LSm8	SPCC1840.10	0.00	0.00	5.24	0.00
Mad1	Spindle assembly checkpoint component mad1	SPBC3D6.04c	-0.23	-0.26	1.27	-2.17
Mad2	Mitotic spindle checkpoint component mad2	SPBC20F10.06	0.00	0.00	5.16	-4.90
Mal3	Microtubule integrity protein mal3	SPAC18G6.15	4.01	-5.29	4.17	0.00
Mcm7	DNA replication licensing factor mcm7	SPBC25D12.03c	0.13	1.04	0.32	-1.47
Mcp6	Meiotic coiled-coil protein 6	SPBC582.06c	0.00	-4.83	1.06	-4.81
Meu18	Meiotic expression up-regulated protein 18	SPBC409.11	0.00	4.76	-3.29	0.00
Mis12	Centromere protein mis12	SPBC409.04c	-4.18	4.83	0.00	0.00
Mis4	Sister chromatid cohesion protein mis4	SPAC31A2.05c	-0.44	-0.49	1.10	-3.27
Mmi1	YTH domain-containing protein mmi1	SPCC736.12c	0.00	-4.62	4.48	0.00

Mst2	Histone acetyltransferase mst2	SPAC17G8.13c	-5.09	4.36	0.00	-3.71
Ndc1	Nuclear envelope protein ndc1	SPAC1786.03	-0.45	-0.83	0.01	1.23
Nsp1	Nucleoporin nsp1	SPAC26A3.15c	0.77	-0.60	1.55	-6.14
Nu120	Nucleoporin nup120	SPBC3B9.16c	-0.06	-0.47	4.64	0.00
Nu131	Nucleoporin nup131	SPBP35G2.06c	0.00	5.07	4.33	0.92
Nu186	Nucleoporin nup186	SPCC290.03c	0.78	-0.23	1.56	-1.43
Nu189	Nucleoporin nup189	SPAC1486.05	0.09	-4.64	1.67	-3.56
Nuf2	Kinetochore protein nuf2	SPAC27F1.04c	0.00	0.00	4.05	0.00
Nup40	Nucleoporin nup40	SPAC19E9.01c	-0.86	-5.00	3.83	0.00
Nup61	Nucleoporin nup61	SPCC18B5.07c	-0.25	-4.31	1.44	-3.68
Nup85	Nucleoporin nup85	SPBC17G9.04c	0.01	0.21	1.18	-3.22
Orc1	Origin recognition complex subunit 1	SPBC29A10.15	0.00	4.60	1.30	0.00
Orc6	Origin recognition complex subunit 6	SPBC2A9.12	4.02	-4.54	-0.70	-6.04
P25	P25 protein	SPAC3C7.14c	-0.02	0.48	1.90	-0.25
Pap	Poly(A) polymerase pla1	SPBC646.04	3.34	-3.93	0.61	-0.05
Pcr1	Transcription factor pcr1	SPAC21E11.03c	0.26	1.01	0.10	1.31
Pdp1	PWWP domain-containing protein 1	SPBC29A3.13	0.36	1.58	-3.87	5.47
Po152	Nucleoporin pom152	SPBC29A10.07	0.84	-0.06	1.70	0.23
Pp12	Serine/threonine-protein phosphatase PP1-2	SPCC31H12.05c	-0.36	1.34	1.31	-4.27
Ppe1	Serine/threonine-protein phosphatase ppe1	SPCC1739.12	-0.74	6.51	0.15	-5.57
Ppme1	Protein phosphatase methylesterase 1	SPCC1739.12	0.31	-0.15	2.58	0.05
Prp16	Pre-mRNA-splicing factor ATP-dependent RNA helicase prp16	SPBC1711.17	3.31	4.15	0.42	0.00
Psb2	Probable proteasome subunit beta type-2	SPAC23D3.07	-0.77	-0.38	5.75	-4.57
Pst3	Paired amphipathic helix protein pst3	SPBC1734.16c	-0.64	-4.05	3.89	-4.32
Rad9	DNA repair protein rad9	SPAC664.07c	-0.51	-0.29	1.60	-1.52
Rcd1	Cell differentiation protein rcd1	SPAC29B12.06c	-0.14	-0.10	1.59	-4.76
Rec10	Meiotic recombination protein rec10	SPAC25G10.04c	0.00	0.00	3.84	0.00
Rec8	Meiotic recombination protein rec8	SPBC29A10.14	-4.08	-4.02	0.00	4.48
Rfa1	Replication factor A protein 1	SPBC660.13c	0.00	0.89	0.00	4.20
Rhp23	UV excision repair protein rhp23	SPBC2D10.12	-0.69	-5.27	2.07	-5.54

Rna1	Ran GTPase-activating protein 1	SPAC22E12.07	0.00	-6.37	4.45	-3.74
Rrp4	Exosome complex component rrp4	SPAC2F7.14c	0.00	4.72	0.00	-3.24
Rrp45	Exosome complex component rrp45	SPCC757.08	-4.19	5.25	0.33	0.00
Rrp46	Exosome complex component rrp46	SPBC115.01c	0.00	4.47	3.76	0.00
Rsc4	Chromatin structure-remodeling complex subunit rsc4	SPBC1734.15	-3.79	4.43	0.00	0.00
Rti1	DNA repair and recombination protein rti1	SPBC119.14	0.00	-4.60	4.41	0.33
Rxt3	Transcriptional regulatory protein rxt3	SPCC1259.07	0.00	0.00	3.77	-4.30
Sad1	Spindle pole body-associated protein sad1	SPBC12D12.01	0.00	5.10	0.04	-5.00
Scc3	Cohesin subunit psc3	SPAC17H9.20	-0.22	0.00	3.60	0.00
Sck2	Serine/threonine-protein kinase sck2	SPAC22E12.14c	0.00	0.00	5.07	-4.85
Sec39	Protein transport protein sec39	SPAC7D4.11c	-1.05	-3.64	1.52	0.00
Seh1	Nucleoporin seh1	SPAC15F9.02	-0.05	1.05	-0.12	-2.19
Sid2	Serine/threonine-protein kinase sid2	SPAC24B11.11c	1.27	0.89	0.55	-4.83
Skp1	Suppressor of kinetochore protein 1	SPBC409.05	-1.07	1.00	1.03	-1.13
Slx8	E3 ubiquitin-protein ligase complex slx8-rfp subunit slx8	SPBC3D6.11c	0.00	7.91	7.33	0.00
Smc1	Structural maintenance of chromosomes protein 1	SPBC29A10.04	0.09	-0.22	2.17	-1.68
Smc3	Structural maintenance of chromosomes protein 3	SPAC10F6.09c	-1.07	-0.88	1.36	0.12
Smd3	Small nuclear ribonucleoprotein Sm D3	SPBC19C2.14	0.25	0.35	1.77	-4.91
Sn114	Pre-mRNA-splicing factor cwf10	SPBC215.12	-0.16	0.28	4.49	-3.65
Snd1	Staphylococcal nuclease domain-containing protein 1	SPCC645.08c	0.23	3.84	0.98	-3.18
Snf22	SWI/SNF chromatin-remodeling complex subunit snf22	SPCC1620.14c	-3.88	0.00	3.35	0.00
Snf5	SWI/SNF chromatin-remodeling complex subunit snf5	SPAC2F7.08c	3.69	0.72	3.62	0.00
Sos7	Kinetochore protein Sos7	SPAPB17E12.06	5.61	0.00	4.19	0.00
Spc24	Kinetochore protein spc24	SPBC336.08	5.30	0.00	-4.32	0.60
Spt16	FACT complex subunit spt16	SPBP8B7.19	-1.51	7.39	-1.42	-1.37
Spt20	SAGA complex subunit spt20	SPAC4D7.10c	3.39	0.47	4.24	0.00
Spt7	Transcriptional activator spt7	SPBC25H2.11c	-2.93	-4.60	2.91	0.00
Ssr2	SWI/SNF and RSC complexes subunit ssr2	SPAC23H3.10	-4.17	-0.39	3.32	-0.48
Sts5	Protein sts5	SPCC16C4.09	0.00	6.79	2.90	0.00
Stu1	Protein peg1	SPAC3G9.12	0.00	-3.68	4.21	-3.20
Sus1	Transcription and mRNA export factor sus1	SPBC6B1.12c	-0.12	-5.25	1.20	-4.35

Swc4	SWR1-complex protein 4	SPAC9G1.13c	-5.17	5.05	-4.74	-0.96
Swi1	Mating-type switching protein swi1	SPBC216.06c	-1.11	-0.50	5.10	-3.12
Swr1	Helicase swr1	SPAC11E3.01c	-1.18	5.92	-2.38	-0.30
Taf10	Transcription initiation factor TFIID subunit 10	SPBC21H7.02	0.15	0.00	6.03	0.00
Taf11	Transcription initiation factor TFIID subunit 11	SPAC1002.04c	3.62	0.00	3.95	-3.97
Taf4	Transcription initiation factor TFIID subunit 4	SPAC23G3.09	-0.83	-0.24	4.13	4.42
Taf6	Transcription initiation factor TFIID subunit 6	SPCC16C4.18c	0.14	1.41	1.53	-0.66
Tip1	Tip elongation protein 1	SPAC3C7.12	-0.89	-5.36	5.30	-4.39
Tlh2	ATP-dependent DNA helicase tlh2	SPAC212.11	0.00	1.29	3.05	0.00
Top2	DNA topoisomerase 2	SPBC1A4.03c	2.90	1.44	4.28	0.00
Tor1	Phosphatidylinositol 3-kinase tor1	SPBC30D10.10c	0.39	0.15	2.18	-3.22
Tra1	Transcription-associated protein 1	SPBP16F5.03c	-0.81	-0.49	1.21	-2.15
Tti1	TEL2-interacting protein 1	SPCC622.13c	0.25	3.36	-4.32	-3.96
Tup12	Transcriptional repressor tup12	SPAC630.14c	-0.63	-4.89	5.82	0.63
Ubr1	E3 ubiquitin-protein ligase ubr1	SPBC19C7.02	0.00	-0.28	3.07	0.00
Xrn2	5-3 exoribonuclease 2	SPAC26A3.12c	0.00	0.00	2.59	2.80
Yaqd	Uncharacterized protein C18G6.13	SPAC18G6.13	-0.39	0.94	5.74	-5.44
Yc93	RanBD1 domain-containing protein C584.03c	SPCC584.03c	0.00	0.00	3.18	-3.57
Ye31	Uncharacterized ABC transporter ATP-binding protein C20G4.01	SPAC20G4.01	-0.24	-5.16	1.41	-3.91
Yee6	Uncharacterized oxidoreductase C19A8.06	SPAC19A8.06	0.01	0.11	1.20	-1.21
Yem8	Uncharacterized protein C29B12.08	SPAC29B12.08	-4.55	0.00	0.00	5.00
Yff4	UPF0616 protein C1687.04	SPAC1687.04	-0.11	0.05	3.51	0.00
Yii3	Uncharacterized transcriptional regulatory protein C139.03	SPAC139.03	-3.87	4.48	0.00	-3.42
Yj44	Uncharacterized membrane protein C70.04c	SPCC70.04c	0.00	5.44	-0.32	1.62
Yjnj	Uncharacterized protein C24B10.19c	SPCC24B10.19c	4.41	0.00	0.08	0.00
Ykcg	Uncharacterized RNA-binding protein C644.16	SPAC644.16	1.29	-0.15	1.07	1.15
Yo48	Putative nucleosome assembly protein C36B7.08c	SPBC36B7.08c	4.69	0.00	-5.20	-4.55
Yoh8	Uncharacterized protein C13E7.08c	SPBC13E7.08c	-4.36	0.00	4.57	0.00
Yon7	PCI domain-containing protein C1105.07c	SPBC1105.07c	5.31	6.73	-0.87	-4.97

Ytm1	Ribosome biogenesis protein ytm1	SPAC890.04c	0.00	-4.24	4.08	-3.20

List shows proteins that were enriched greater than two-fold at one or more time points of meiosis and that are annotated in the genome database as being nuclear, chromatin associated, or both. Ratio values (note log2 scale) indicate greater abundance in hotspot (positive numbers) or basal control (negative numbers) MiniCs, using sum of iBAQ abundance values from three biological replicates except for the 1 hour time point, for which there were two replicates.
